# Genetic Algorithm-Based Data-Driven Process Selection System for Additive Manufacturing in Industry 4.0

**DOI:** 10.3390/ma17184544

**Published:** 2024-09-16

**Authors:** Bader Alwomi Aljabali, Joseph Shelton, Salil Desai

**Affiliations:** 1 Department of Industrial & Systems Engineering, College of Engineering, North Carolina A & T State University, Greensboro, NC 27411, USA; bmalwoim@aggies.ncat.edu; 2 Department of Computer Science, College of Engineering and Technology, Virginia State University, Petersburg, VA 23806, USA; jshelton@vsu.edu; 3 Center for Excellence in Product Design and Advanced Manufacturing, North Carolina A & T State University, Greensboro, NC 27411, USA

**Keywords:** genetic algorithm, design for additive manufacturing, expert system, Industry 4.0

## Abstract

Additive manufacturing (AM) has impacted the manufacturing of complex three-dimensional objects in multiple materials for a wide array of applications. However, additive manufacturing, as an upcoming field, lacks automated and specific design rules for different AM processes. Moreover, the selection of specific AM processes for different geometries requires expert knowledge, which is difficult to replicate. An automated and data-driven system is needed that can capture the AM expert knowledge base and apply it to 3D-printed parts to avoid manufacturability issues. This research aims to develop a data-driven system for AM process selection within the design for additive manufacturing (DFAM) framework for Industry 4.0. A Genetic and Evolutionary Feature Weighting technique was optimized using 3D CAD data as an input to identify the optimal AM technique based on several requirements and constraints. A two-stage model was developed wherein the stage 1 model displayed average accuracies of 70% and the stage 2 model showed higher average accuracies of up to 97.33% based on quantitative feature labeling and augmentation of the datasets. The steady-state genetic algorithm (SSGA) was determined to be the most effective algorithm after benchmarking against estimation of distribution algorithm (EDA) and particle swarm optimization (PSO) algorithms, respectively. The output of this system leads to the identification of optimal AM processes for manufacturing 3D objects. This paper presents an automated design for an additive manufacturing system that is accurate and can be extended to other 3D-printing processes.

## 1. Introduction

Additive Manufacturing (AM) is poised to be part of the industrial revolution given its versatility to fabricate free-form designs in a variety of materials [1,2,3,4]. In recent years, the AM field has evolved from rapid prototyping to mainstream manufacturing due to the capability of building complex three-dimensional freeform features with multiple materials [5,6,7,8]. Additive manufacturing, popularly called “3D Printing” is now being implemented in fields ranging from biomedical devices [9,10,11], semiconductor electronics [12,13], and energy devices [14,15,16] to the construction industry [17,18]. Several additive manufacturing technologies have mushroomed, each with their own unique process capabilities [19,20,21]. The parts to be fabricated on commercial 3D printers need appropriate evaluation from AM experts for optimal usage of material and machine capability [22]. However, it takes longer spans of time and working knowledge of different AM technologies to be proficient in this task. Moreover, access to such specialized resources and biases in expert opinion can hamper their implementation. Since AM is still in its evolving stages of its industrial application [23], off-the-shelf AM process guidelines, test data, and machine capability know-how are limited, thereby restricting the usage of AM technologies to subject matter experts [24]. Thus, it is imperative that a generic system be developed that can assist novice designers and AM enthusiasts in finding the optimal solution/technique for their tasks [25,26]. Consequently, such a generic system would lead to effective maximization of the benefits and widespread use of AM technologies. Even though AM technologies exempt designers from the myriad constraints often encountered in traditional manufacturing, they require several design rules and constraints that must be adhered to. As aforementioned, the current structure of AM lacks systematic guidance to encapsulate AM expert know-how, test data, and design rules [26,27]. To tackle this issue, ASTM International aims to publish a guide to standardize the work process and simplify the creation of parts with additive manufacturing [28]. Without such a guide, engineers and researchers in the field will be faced with the problem of choosing appropriate AM processes and material specifications for their applications. The ASTM Committee F42 has already produced several standards relating to AM [29].

With the rise of automation, expert systems have gained significant popularity in manufacturing and material processing, leading to more efficient processes through automatic control mechanisms [30,31]. These systems are powered by artificial intelligence (AI) and heuristic algorithms, enabling autonomous decision-making [32,33]. For instance, Nagarajan et al. [34] developed a novel knowledge-based artificial neural network (KB-ANN) by combining dimensional analysis conceptual modeling (DACM) with classical ANNs. Their approach incorporated existing literature and expert knowledge of additive manufacturing (AM) processes to design a KB-ANN model. This hybrid network includes topological zones informed by process knowledge and zones where knowledge gaps are filled using traditional ANNs. Similarly, Dwivedi, Rajeev, and Radovan Kovacevic created an expert system for laser-based multi-directional metal deposition (LBMDMD) to automate previously manual process planning steps. Their system utilized an AI-based computer-aided design and manufacturing platform to fabricate parts directly from digital models [35]. Additionally, a rapid manufacturing (RM) system was developed to assist designers in selecting optimal production parameters based on initial design requirements [36]. The team introduced a computer-aided system (RMADS) that utilized fuzzy inference, relational databases, and rule-based decision-making. This rule-based expert system recommended rapid prototyping (RP) systems and their specifications through an interactive question-and-answer session with the user [31]. Fountas et al. explored [37] the optimization of fused deposition modeling (FDM) using a variety of single and multi-objective evolutionary algorithms. Their research tested algorithms such as Dragonfly, Ant–Lion, and Grey Wolf in optimizing critical parameters like compressive strength, sliding wear, dimensional error, and build time. The results highlighted the effectiveness of these algorithms in improving the performance of FDM processes and validated the “No-Free Lunch” theorem, which suggests that no single algorithm performs optimally across all optimization problems. In a second study, Fountas et al. [38] also made significant contributions to the field by introducing a Virus-Evolutionary Genetic Algorithm (VEGA) for optimizing selective laser sintering/melting (SLS/SLM) processes. Their research demonstrated VEGA’s superior performance over other algorithms such as Grey Wolf and Ant–Lion, particularly in solving multi-objective optimization problems. VEGA’s ability to optimize critical parameters like density, hardness, and tensile strength for materials like Ti6Al4V and L316 stainless steel underscores its potential to enhance the efficiency and quality of additive manufacturing operations. Despite these advancements, the development of a versatile expert system that can operate across multiple processes, materials, and application domains remains an area in need of further research.

This research aims to develop a data-driven expert system for the optimal selection of additive manufacturing (AM) processes based on CAD data and user-defined preferences. To achieve this, we implemented a genetic algorithm (GA) for classifying AM techniques using the parametric data of 3D-printed parts. GAs, which are inspired by Darwinian principles of natural selection [39], are well-suited for solving complex classification problems in datasets with intricate, confounding relationships among variables [40,41,42,43]. Previous research has demonstrated the efficacy of GAs for classification tasks, particularly when combined with neural networks for problems such as geographical image classification, where GAs optimized the neural network architecture for enhanced performance [23,44,45]. In the context of AM, GAs have been employed in a variety of optimization tasks. For instance, Ashan and Khoda [46] utilized a GA to optimize build orientation and tool path deposition, reducing the complexity of AM production. Similarly, Zhishuai et al. [47] applied a GPU-based parallel GA to minimize build time, surface quality degradation, and support structure requirements in a multi-objective optimization problem. Deka and Behda [48] further extended the application of GAs to improve productivity through part separation optimization, while others have used GAs to refine the geometry and topology of internal and external support structures [49].

In this study, the GA was selected for its ability to quantify solutions based on a fitness function, which allowed for flexibility in adapting to user preferences and system requirements. While neural networks are traditionally more suited for pattern recognition tasks [22], the GA proved to be an appropriate choice for designing a classification system for AM process selection. Specifically, we implemented a genetic and evolutionary feature weighting (GEFeW) algorithm [50] to account for all criteria necessary for manufacturing a 3D-printed object. To the best of our knowledge, there has been limited exploration of GA application in AM process selection, making this study a novel contribution. In this research, the weights for each criterion were evaluated to identify an unknown print object by comparing it to a database of other known objects labeled with the optimal AM technique. The trained weights were tested on a dataset of 3D-printed objects to determine their effectiveness. The data-driven AM process selection methodology developed in this research serves as the basis for a new Design for Additive Manufacturing paradigm. 

Contributions of this Research

The key contributions of this paper are:

- Identifying the need for a versatile AM expert system capable of selecting the optimal additive manufacturing technique for 3D printed parts across different processes, materials, and application domains, underscoring this as a key area for future research.
- The development of a novel data-driven expert system for the optimal selection of additive manufacturing (AM) processes, based on CAD data and user preferences.
- The implementation of a genetic algorithm (GA) to classify AM techniques by analyzing parametric data, addressing complex classification challenges specific to AM.
- Introduction of the genetic and evolutionary feature weighting (GEFeW) algorithm, which systematically evaluates and weighs criteria for AM process selection, providing a flexible and adaptive optimization approach.

## 2. Methodology

**Figure 1:**
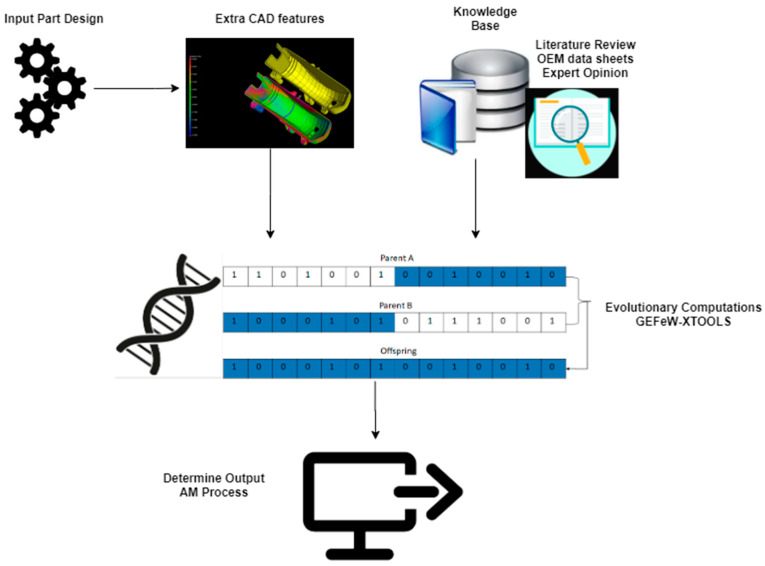
Framework for a data-driven process selection system for additive manufacturing.

This research aims to develop a heuristic model that can determine the preferred additive manufacturing (AM) technique for a 3D-printed part. Figure 1 shows a schematic of the DFAM methodology, which consists of the input part characteristics, knowledge base, genetic algorithm engine, and output identification of an optimal AM technique. In this methodology, the input part characteristics were extracted from both the part CAD file and user requirements. A knowledgebase was compiled from OEM datasheets to determine AM process specifications. Datasets were populated by combining inputs from the CAD extraction, user requirements, and knowledgebase. Evolutionary algorithms were implemented on the datasets to generate weights on different features for each part design. A two-stage Metamodel was developed using the genetic algorithm based on input vector specification and data augmentation. The developed genetic algorithm engine had high prediction accuracy for identifying optimal AM processes for different part designs. 

User preferences for the material types were obtained, as each AM technique can be built with a limited set of material combinations. In addition, other specified components, such as desired build speed, surface finish, and feature resolution, were recorded. These inputs were used to evaluate potential AM techniques for building each part based on the machine’s capabilities.

**Figure 2:**
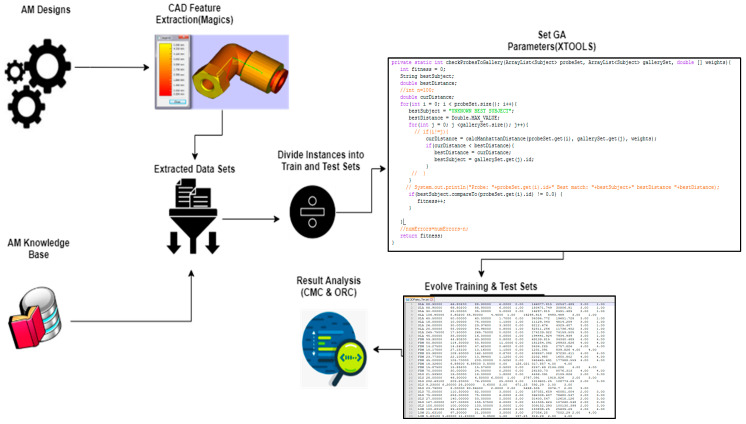
Flowchart for a data-driven process selection system for additive manufacturing.

As shown in Figure 2, the flow of steps that determined the optimal AM technique started with extracting all the information needed from the part CAD file, converting it to .stl or .amf formats, and creating the datasheet file. The user preferences were recorded, and part designs were classified based on inputs from the knowledge base. Then, the data on file were divided into instances such as the training set and the test set, to assign the appropriate GA parameters. Also, the eXploratory tool set for the optimization of launch and space systems (XTOOLSS) software [51] was used to run the genetic algorithm. It evolved solutions to training sets to be applied to test sets to finalize the results.

### 2.1. Generating Inputs to the Framework

**Figure 3:**
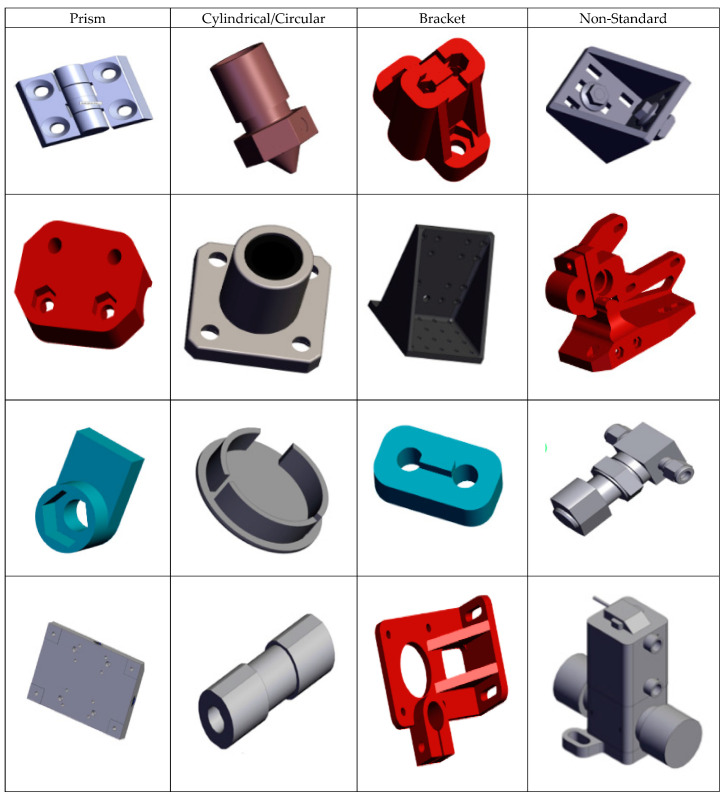
Sample parts with different topologies as inputs for the GA algorithm.

**Figure 4:**
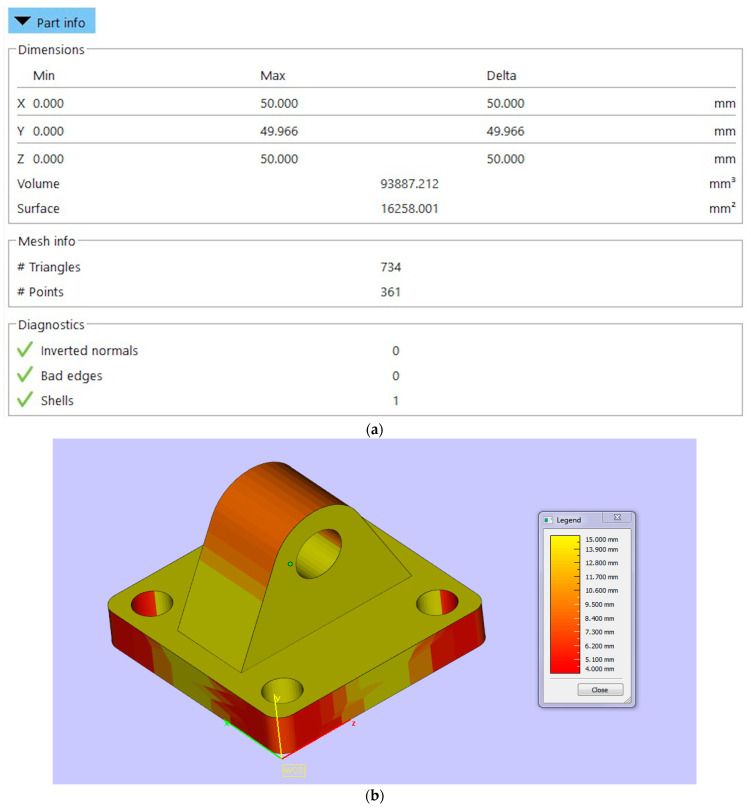
(**a**) Dimensional, volumetric, and surface area data being extracted from part design and (**b**) minimum part thickness evaluated based on quantifying the variation in thickness for the part geometry.

To generate inputs specific to the framework shown in Figure 1, 3D CAD models of parts (see Figure 3) were downloaded from an online CAD repository—3D Content Central [52]. The part geometries represented different parts to be fabricated using additive manufacturing. These parts were converted to .stl or .amf format and analyzed for volumetric and surface area data using the Materialize Magics© software suite [53]. Figure 4a shows the XYZ dimensions, volumetric and surface area data being extracted for this part. The minimum part thickness was evaluated based on quantifying the variation in thickness for the part geometry as shown in Figure 4b.

The data collected using the above steps were populated within a datasheet to be fed to the GA. Nine input variables were defined for capturing the part information. The dimensional information was recorded in millimeters. Table 1 shows the sample input and output features for training the GA model. 

The genetic algorithm was trained with instances representing the features that described a model to be printed. The features listed below (thickness, dimension, surface finish, etc.) were initially placed in a datasheet where each row denoted an object, as shown in Table 1. The material keys include 1—Polymer, 2–Metal, 3—Ceramic, 4—Paper, 5—Composite. The objects were classified based on the best technique to print them. As an example, set number 11 (SLA2 1 299 130 213 8279310 8.5) represented an object with material using plastic (where plastic is 1, metal is 2, and so on) with a length of 299 mm, a width of 130 mm, a height of 213 mm, an object volume of 8,279,310 mm^3^, and a minimum thickness of 8.5 mm for the part. The best technique to print this object was determined to be stereolithography.

### 2.2. Knowledge Base

A knowledge database was created by populating information from a literature review, AM manufacturer specifications, and experimental and computational models for different AM processes [54,55,56,57,58]. As shown in Table 2, examples of AM technique specifications were introduced for knowledge base such as surface finish, minimum thickness, build volume (XYZ), resoultion, material, and build speed. 

### 2.3. Genetic and Evolutionary Weighting for AM Classification

Advancements in technology, modeling, and simulation tools are vital in engineering design to improve the speed and enhance the cost efficiency of the design cycle. Evolutionary computations, such as genetic algorithms, can be used to optimize solutions for any defined problem. With increasing design parameters, it becomes very difficult to obtain optimal solutions as systems become more complex. Genetic algorithms (GAs) operate on the principle of natural selection (survival of the fittest) to search the population. GAs were made relatively easier to use by the development of software with user-friendly interfaces. This enables engineers to solve complex optimization problems with minimal understanding of the system (mainly the system parameters being optimized and their role in system operation). 

The eXploratory tool set for the optimization of launch and space systems (XTOOLSS) source code [51,59] was used to execute the genetic algorithm. Table 3 shows the parameters used for the stage 1 and stage 2 datasets. For each algorithm, there were 30 runs performed on the training set. For normally distributed data, approximately 30 observations were needed to have reasonably short confidence bounds on the variance estimate. The best solution from each run was applied to a test set. 

XTOOLSS [51] allows the user to implement the fitness function in java. The problem is, ‘if a person has an object that they want to 3D print, what is the best AM technique to use?’ To come up with the solution, consider all the information that the user could possibly know. A training set was constructed that contained instances that were each represented by a set of nine variables described earlier. The Genetic and Evolutionary Feature Weighting (GEFeW) [50] technique was used to evolve a set of weights, denoted as a vector of feature weights (FWs). Wi = {wi, 0, wi, 1,…, wi,n − 1} represents the weighting values for each set of weights. The fitness f_i_ = 10 ε represents the fitness of any evolved FW, where ε denotes the number of errors that an FW obtains on a dataset.

Fitness = Minimize {10 × the number of incorrect instances}(1)

The Fitness formula explains a minimizing evaluation function that seeks to attain solutions close to a fitness value of zero. Offspring FWs are created based on the previously selected parents. The offspring are then evaluated and assigned fitness based on their performance on the training set. After the offspring is evaluated, a new population is then formed by replacing the worst individual in the population with the offspring. 

For this problem, identification is used to classify the best technique to use for a job. Identification is the process of comparing different instances to one another and determining whether the system can determine the identity of an instance. Instances are classified based on the best technique for that instance to use for 3D printing. Given the problem definition, the ‘solution’ is represented as a set of weights to apply to instances as they are being compared. The weights were trained on the training set. The fitness is the number of instances incorrectly classified in the training set. The absolute best fitness solution can have from the fitness function is 0.0, while the worst is the number of instances in the training set.

The best weights (that were optimized on the training set) were generalized on some test sets of mutually exclusive instances to gauge the performance of the evolved weights. The test set was used to test how accurate the evolved weights were in classifying unknown objects by the optimal printing technique. An unknown portion of the test set was compared to the remaining portions of the test set via identification. The weights were applied to all instances during identification. The output of the model was defined as the accuracy of identifying the unknown portions by technique. GEFeW was chosen as the technique for classification due to its low computational cost compared to other techniques such as neural network classifiers or random forest classifiers for feature selection [50]. 

## 3. Results and Discussion

### 3.1. CAD Data Extraction and Knowledge Base

To demonstrate the feasibility of the framework, a two-stage case study was implemented. CAD part files were downloaded from the online CAD database, where part types represented different part geometries used in real-life examples. These included prismatic, cylindrical, and combinatorial features and topographies such as brackets, couplings, housings, etc. A genetic algorithm was used to evolve weights for the AM technique classification problem, as seen in the flow chart in Figure 2. Two different datasets and input attributes were used. Stage 1 comprised 100-part designs with coded keys for user-specified attributes, whereas stage 2 comprised 300-part designs with actual numerical values for all attributes. 

#### 3.1.1. Stage 1 GA with 100-Part Designs

In stage 1, the datasheet was populated with 100 print objects. In total, 50% of the instances composed the training set, while the other 50% composed the testing set. Each part’s information was represented by nine features. These include material type, length (x), width (y), height (z), object volume, minimum wall thickness, surface finish, build speed, and feature resolution. The dimensions were measured in millimeters. Surface finish, build speed, and feature resolution were coded as three-level keys as shown in Table 5. The material type feature was numerically coded as five types of materials as shown in Table 4. There were 10 AM techniques used for classification: Stereolithography (SLA1, SLA2), Fused Deposition Modelling (FDM1, FDM2), Selective Laser Sintering (SLS1, SLS2), 3D Printing (3DP1, 3DP2), Laminated Object Manufacturing (LOM1 and LOM2). The larger dimensions were labeled technique_1, and the smaller-sized objects were labeled technique_2. The two sub-classifications for the AM machines were labeled to represent differences in AM process capability in terms of build volume and build speed. Each classification had 10 instances. Instances were labeled appropriately as the best technique to use by consulting experts in the AM field. The coded keys for the AM technique and part feature attributes are shown in Table 4. 

There were 30 runs of GEFeW performed on the AM dataset, resulting in 30 optimized sets of weights on the training set. Table 5 shows the average identification accuracy of the 30 weights on the test sets as well as the best accuracy. These were compared to a baseline approach. The baseline approach uses the Manhattan distance metric to identify different instances with no weights for features.

The results show that the Genetic and Evolutionary Feature Weighting (GEFeW) approach has a better performance than the baseline approach regarding the identification of the average and the best.

**Figure 5:**
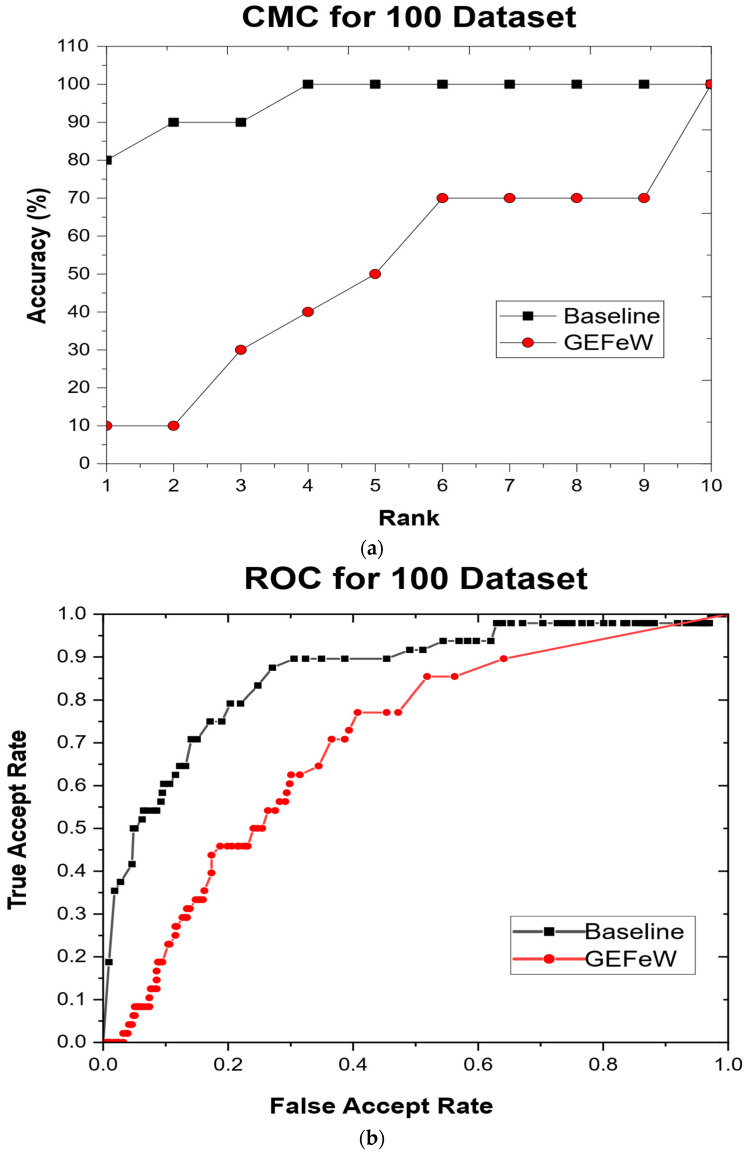
(**a**) Cumulative match characteristic (CMC) curves and (**b**) receiver operator characteristic (ROC) for stage 1 (100 dataset).

Figure 5a,b show the cumulative match characteristic (CMC) curves and receiver operator characteristic (ROC) curves for the baseline approach and GEFeW applied on the test set, respectively. The CMC curve (Figure 5a) denotes the rank accuracy of both techniques. The rank represents the closeness of identification; therefore, a rank of two means that an approach correctly identified an object within the two closest matches. GEFeW has a rank 1 accuracy of 80% and achieved a 100% accuracy at rank 4, whereas the baseline approach did not achieve 100% until rank 10 and had an initial accuracy of 10%. The ROC curve (Figure 5b) denotes the verification performance of both GEFeW and the baseline on the test set. Verification is a representation of how similar two objects are to each other. The ROC curve plots the true accept rate (TAR) against the false accept rate (FAR). TAR represents the similarity of all instances compared to their respective counterparts, whereas FAR represents the instances compared to other, nonmatching, instances. The results show that the GEFeW technique has a superior TAR to FAR ratio, while the baseline has poor performance. 

There were 30 runs of GEFeW performed on the AM dataset, resulting in 30 optimized sets of weights on the training set. The 30 weights were generalized on a test set of instances mutually exclusive from the training set, and the performance was recorded. The GEFeW approach had a significantly better performance than the baseline (no weights), suggesting that weighting features are necessary for correct AM classification. 

Table 6 shows the average weight for each feature; each weight signifies the importance of that feature for classification. The highest-weighted features are material type, build speed, surface finish, and feature resolution. The features with the lowest weights were the XYZ dimensions, build volume and minimum thickness. Though there were AM techniques that were better for certain-sized objects, overall, the user preferences such as surface finish, build speed, and feature resolution seem to contribute the most towards determining the best AM technique to use. The dimension-related weights seem to have the lowest weights, but when GEFeW was run on a dataset with these features removed, the overall classification accuracy decreased. More specifically, the average accuracy decreased from 70% to 64% when these features were eliminated. This result suggests that, though the significance of the dimension features is minimal, the inclusion of these features is important. 

Table 7 shows the frequency of correct AM classifications for each instance when applying the 30 sets of weights on the test set. The classification labeled 3DP2 was misclassified most often (not getting any correct), while the LOM instances were correctly classified for all 30 weights. There are several instances where, among the same set, one set had nearly all 30 weights correctly classify the instance (FDM1, SLA1, 3DP1) while the other set only had one or fewer correctly classified (FDM2, SLA2, 3DP2). The FDM2 instances were incorrectly classified as SLS2, the 3DP1 instances were mislabeled as 3DP2, and the SLA2 instances were misclassified as FDM2 in most of the runs. A comparison of the testing data shows that the SLA2 and FDM2 instances have similar build speeds, feature resolutions, and material features. Between the FDM1 and FDM2 sets, the dimensions were vast enough to distinguish between the two sets. The dimension sizes are also similar for the SLS 1 and 2 sets, suggesting that more attention must be paid to the user preference features when doing classification. The 3DP1 dataset had a higher classification than 3DP2, due to the dimension sizes as well. The Manhattan distance metric was used, but normalization may be necessary to prevent any large difference in similarity scores.

#### 3.1.2. Stage 2 GA with 300-Part Designs

The AM classification accuracies for part designs in stage 1 were low for reliable AM process selection prediction. This problem was addressed by increasing the datasets to 300-part designs and quantifying the coded keys for specific attributes (surface finish, build speed, and feature resolution). The 300 datasets were divided into 200 training and 100 test datasets. Each AM sub-classification was assigned an equal number of part designs in the training set. The user input features (surface finish, build speed, and feature resolution) were quantified as numerical range values for each AM process based on information gathered from OEM data sheets and expert feedback [60,61,62]. The ranges for surface finish, build speed, and feature resolution are shown in Table 8 for different AM processes. 

The instances were labeled with the optimal technique for the job. The design rules obtained through the literature review helped determine what would be included in the instances. Therefore, if an instance was labeled as selective laser sintering (SLS), it could have used specific materials and would have a certain build speed associated with it [56]. Five additive manufacturing techniques were further subdivided into two categories based on the differences in build volume [63]. A total of 300 datasets were split into 200 for training and 100 for testing sets, respectively. This dataset was used to execute the genetic algorithm with nine (9) part attributes (input variables).

The steady generational genetic algorithm (SSGA), estimation of distribution algorithm (EDA) and a particle swarm optimization (PSO) technique were selected due to their distinct algorithmic properties and proven effectiveness in optimization tasks, particularly in complex, multi-dimensional search spaces encountered in additive manufacturing. In this research, SSGA demonstrated its ability to maintain genetic diversity and avoid premature convergence, which is critical in finding optimal solutions in highly non-linear and multi-modal landscapes typical in additive manufacturing. SSGA’s steady-state nature allows for a more controlled and gradual evolution of the population, providing a balance between exploration and exploitation of the search space.

EDA was included because it represents a probabilistic model-based approach, which differs significantly from the population-based search mechanisms of genetic algorithms and PSO. EDA constructs and updates a probabilistic model of promising solutions, enabling it to capture and exploit the underlying structure of the problem space. This characteristic is particularly useful when the problem domain has inherent probabilistic relationships between variables such as build volume and build speed, as is often the case in additive manufacturing processes. 

PSO was selected due to its simplicity, ease of implementation, and effectiveness in handling continuous optimization problems. It mimics the social behavior of birds flocking or fish schooling, making it well-suited for searching large, continuous spaces. PSO’s reliance on the collective behavior of particles to explore the search space can be advantageous for global optimization, which is essential in additive manufacturing where parameter interactions are complex and non-linear. In our research, the relationship between material type, resolution and surface finish depends on several factors such as build speed and layer height. 

By comparing these three distinct techniques, this research aimed to assess the strengths and limitations of each approach in the context of additive manufacturing, ultimately demonstrating the robustness and superior performance of SSGA in this application. The diversity in the selected algorithms ensured a comprehensive evaluation of different optimization strategies, providing valuable insights into the most effective methods for optimizing additive manufacturing parameters.

Given the low dimension space of the problem (selecting parameters to optimize the AM process), it was determined that the computational complexity of the environment used to execute the genetic algorithm is not the key aspect of this research, as the created solution from XTOOLSS would be generally used and the time of creation will not impact future uses of the generated parameters.

Several researchers have applied machine learning and artificial intelligence models to AM process modeling. Aminzadeh and Kurfess implement a Bayesian classification in powder-bed AM using visual camera images [64] for quality monitoring. Zhu et al. [65] provided a prescriptive deviation modeling methodology to control geometrical variations in AM. Further, Wu et al. [66] utilized an acoustic emission sensor coupled with a hidden semi-Markov model to identify failure states in fused deposition modeling. Olowe et al. [67] implemented a harmonic-percussive source separation technique along with eight different machine learning algorithms for predictive quality control in AM. Yang et al. [68,69] incorporated a rheological process parameter study to identify optimal factors in slurry-based 3D printing processes. Mehrpouya et al. [70] applied neural networks to generate a nonlinear map to identify optimal operational parameters in shape memory alloys using AM. Hoenig et al. [71] discussed the need to integrate explainable artificial intelligence (XAI) with cyber-physical systems such as additive manufacturing. In spite of these attempts to improve AM processes, there exists no holistic AI model that can assist in translating input product designs to specific AM processes based on design and user requirements. Our results described herein clearly demonstrate the potential of SSGA to address process selection issues in AM.

**Figure 6:**
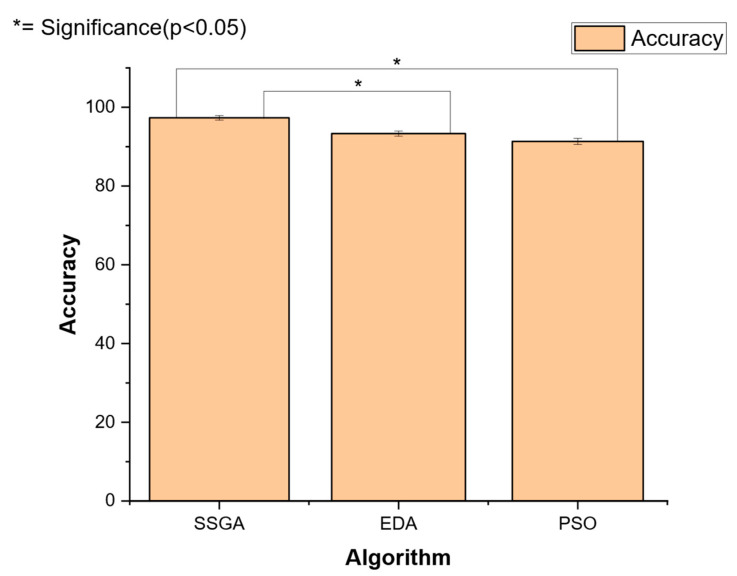
Comparative statistical analysis of algorithms for additive manufacturing process selection.

Each run was executed for 8000 generations. A steady-state genetic algorithm (SSGA) was determined to be the most effective algorithm after preliminary testing. A preliminary experiment was performed on a training set of 200 instances (40 instances per technique). The recognition accuracy of the steady generational genetic algorithm (SSGA) was shown to be significantly superior when compared to an estimation of distribution algorithm (EDA) and a particle swarm optimization (PSO) technique. The EDA and PSO techniques were run for 8000 function evaluations, with a population size of 20 for each algorithm. To verify the observation that SSGA outperformed EDA and PSO, a factorial analysis of variance (ANOVA) was performed. Table 9 shows the ANOVA that was carried out with an ⍺ = 0.05, and a resulting *p*-value of 0.0033, suggesting a significant difference among the three groups of data (SSGA, EDA, and PSO). Figure 6 shows the comparative analysis of the three algorithms. A significant difference was shown suggesting SSGA as the best performance, which had an average performance of 97.33%. The average PSO accuracy was 91.33%, and the average accuracy of the EDA was 93.33%. 

The SSGA for the experimental results was implemented with a population size of 30, a Gaussian mutation rate of 0.2, and uniform crossover and binary tournament selection. The best solution from each run was applied to a test set. Each instance within the datasets was an object represented by its dimensions, wall thickness, volume, material, preferred build speed, and preferred surface finish. 

**Figure 7:**
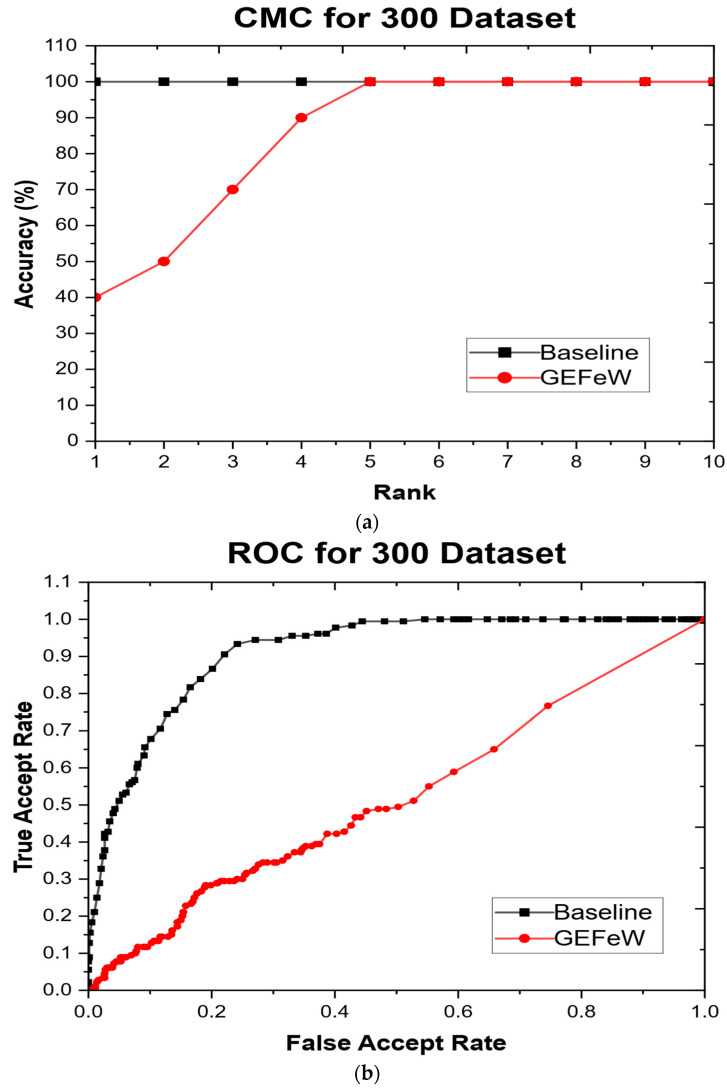
(**a**) Cumulative match characteristic (CMC) curves and (**b**) receiver operator characteristic (ROC) for stage 2 (300 dataset).

Figure 7a,b show the cumulative match characteristic (CMC), curves and receiver operator characteristic (ROC) curves for the baseline approach and GEFeW applied to the test set. The CMC curve denotes the rank accuracy of both techniques. The rank represents the closeness of identification, so a rank of two means that an approach correctly identified an object within the two closest matches. GEFeW has a rank 1 accuracy of 100%, whereas the baseline approach does not achieve 100% until rank 5 and had an initial accuracy of 40%. The ROC curve denotes the verification performance of both GEFeW and the baseline on the test set. Verification is a representation of how similar two objects are to each other. The ROC curve plots the true accept rate (TAR) against the false accept rate (FAR). TAR represents the similarity of all instances compared to their respective counterparts, whereas FAR represents the instances compared to other, nonmatching instances. The results show that the GEFeW technique has a superior TAR to FAR ratio, while the baseline has poor performance.

There were 30 runs of GEFeW performed on the AM dataset, resulting in 30 optimized sets of weights on the training set. The 30 weights were generalized on a test set of instances mutually exclusive from the training set, and the performance was recorded. The GEFeW approach had a significantly better performance than the baseline (no weights), suggesting that weighting features are necessary for correct AM classification. Table 10 shows the average weight for each feature; each weight signifies the importance of that feature for classification. The highest-weighted features were build speed, build volume, and surface finish.

The features with the lowest weights were the minimum thickness, XYZ dimensions, and feature resolution. Though there are techniques that are better for certain-sized objects, overall, object preference seems to contribute the most towards determining the best AM technique to use. The dimension-related weights seem to have the lowest weights, but when GEFeW was run on a dataset with these features removed, the overall classification accuracy decreased. This result suggests that, though the significance of the dimension features is minimal, the inclusion of these features is important overall.

Table 11 shows the frequency of correct classifications for each instance when applying the 30 sets to weights on the test set. The instances labeled 3DP2 were misclassified most often (not getting some correct), while the LOM instances were correctly classified for all 30 weights. Both FDM and SLA processes had 100% prediction accuracy for part classification. The SLS1 had accurate predictions; however, the SLS2 process missed correctly predicting two-part instances. Between the FDM1 and FDM2 sets, the dimensions were vast enough to distinguish between the two sets. The dimension sizes are also similar for the SLS 1 and 2 sets, suggesting that more attention must be paid to the user preference features when doing classification. The 3DP1 dataset had a higher classification than 3DP2, due to the dimension sizes as well. The Manhattan distance metric was used but normalization may be necessary to prevent any large difference in similarity scores. 

In stage 2, the inclusion of quantitative datasets for each feature as well as augmenting data to 300 printable objects had a positive influence on the prediction accuracies of the GA algorithm. The frequency of correct classification for each AM process subset increased substantially as compared to stage 1 (100 datasets). In particular, the FDM2, SLS1 and 3DP2 processes had a dramatic increase in classification accuracy from 1 or 0 in 30 instances (shown in Table 7) to 30 of 30 (Table 11) object classifications accurately identified for stage 2. Figure 6 clearly shows that the 300 datasets had a superior ROC performance reaching 90% accuracy far earlier as compared to Figure 5 for the 100 datasets. Thus, it can be estimated that providing specific quantitative feature instances and a higher number of datasets provides a higher discriminating power of the genetic algorithm. The steady-state generational genetic algorithm (SSGA) provided a 97% average accuracy as benchmarked against the estimation of distribution algorithm (EDA) and a particle swarm optimization (PSO) technique. This research demonstrates an automated additive manufacturing selection system wherein parts can be accurately assigned to respective 3D printing processes which would require expert guidance that is acquired over several years in the AM field. Its practical utility can benefit additive manufacturing service bureaus to assign CAD designs to different AM machines on their network with minimal human intervention. 

## 4. Limitations and Future Work

While this research presents a promising data-driven system for identifying optimal additive manufacturing (AM) processes based on CAD data and user requirements, one key limitation is the relatively small size and variety of the dataset used for training and testing the genetic and evolutionary feature weighting (GEFeW) algorithm. The limited dataset may have affected the robustness of the model, particularly in the consistent misclassification of SLS2 and 3DP2 labeled instances. In terms of future work, the first focus is expanding the dataset to include a broader variety of instances, which will further improve the system’s performance and ensure its adaptability to a wider range of applications. Future research will also explore combining multiple classifiers with the GEFeW approach to enhance classification accuracy further. This will establish a more comprehensive framework for identifying optimal printing techniques. The ultimate aim is to evolve this work into a comprehensive Design for Additive Manufacturing (DFAM) system that can define precise design guidelines tailored to the specific AM process used, thereby enabling more efficient and automated decision-making in 3D printing. In addition, this proof-of-concept methodology will be interfaced with industry-based CAD software packages and commercial 3D printing portals to demonstrate its practical impact. 

## 5. Conclusions

A data-driven system for identifying preferred additive manufacturing processes is presented based on input part CAD data and user requirements. The genetic and evolutionary feature weighting (GEFeW) approach was used to classify the optimal AM technique based on several input features including part dimensions, build speed, surface finish, and material specification. The results were obtained when identifying a set of print object instances in a database of instances with different preferable techniques. The results showed that the GEFeW technique was superior to the generic distance metric approach with no weights regarding identification. The verification results also show superior performance for GEFeW compared to the baseline, though the fitness function was optimizing identification. Out of the 30 runs, the SLS2 and 3DP2 labeled instances were the most consistently misclassified, while other objects were consistently classified correctly. This research shows promise as a new method for determining optimal printing techniques for 3D-printed objects over the current practice of consulting an AM expert. This work will be extended by creating larger datasets, with a greater variety of instances for optimal training. Different classifiers can also be combined for improved performance. GEFeW was purely optimized on a training set, and overfitting was avoided to have a winning performance on the mutually exclusive test set. For larger datasets, a validation set of mutually exclusive instances will be introduced during the training process. During the training process, masks will be trained on the training set, but the trained instances will be applied to the validation set, and the best-performing mask will be recorded over time. This will ensure that no mask is overfitted to only seek the local optimal solution. This research establishes the foundation for the development of a Design for Additive Manufacturing (DFAM) system that can be extended to specify design rules based on the underlying AM process. 

## Figures and Tables

**Table 1:** Sample datasheet contains some features classified based on best techniques.

OUTPUT		INPUT Variables							Units (mm)	
		Features Extracted from AMF and STL						GUI Specified Features		
Set	AM Technique	Material	Length (X) (mm)	Width (Y) (mm)	Height (Z) (mm)	Object Volume (mm^3^)	Minimum Thickness (mm)	Surface Finish (µm)	Build Speed (mm/h)	Feature Resolution (µm)
1	LOM2	4	3000	3000	3000	27,000,000,000	30	3	3	3
2	LOM2	4	1510.4	755	1030	1,174,562,560	2.5	3	3	3
3	SLS1	3	59.538	39.642	20.015	47,239.511	0.3	2	2	2
4	LOM1	4	100.631	99.2	24.2	241,578.8038	2.8	2	2	2
5	LOM1	1	66	50	60	198,000	3	2	2	2
6	LOM1	4	88.9	157.15	6.35	88,713.53225	5.5	2	2	2
7	FDM2	1	228.6	228.6	165.1	8,627,789.196	9	2	2	2
8	FDM2	1	152.4	151.724	203.2	4,698,540.28	8	2	2	2
9	FDM1	1	54.247	24.581	6.924	9232.77669	1	2	2	2
10	FDM1	1	40	20.005	11.09	8874.218	0.35	2	2	2
11	SLA2	1	299	130	213	8,279,310	8.5	2	2	2
12	SLS2	3	150	120	150	2,700,000	3.65	2	2	2

**Table 2:** Specifications of AM processes populated as part of knowledge base.

No.	AM Technique	Build Volume (mm^3^)	Build Speed (mm/hr)	Surface Finish (µm)	Material	Resolution (µm)	Minimum Thickness (mm)
1	FDM	(355 × 305 × 305)	7–43	150–300	Polymers	200–400	0.33
2	SLA	(736 × 635 × 533)	7–30	80–130	Polymers	35–100	0.1
3	SLS	(381 × 330 × 460)	25–60	125–250	Metal	60–150	0.10
4	3DP	(914 × 610 × 914)	20–50	180–300	PLA, ABS	50–180	0.02
6	LOM	(381 × 254 × 355)	40–150	200–500	Paper, plastic, and some sheet metals	100–300 Paper, 150–300 Plastic	0.1–0.19

**Table 3:** GA parameters used in stage 1 and stage 2.

Parameters	100 Datasets	300 Datasets
**Population size**	20	30
**Total evaluations**	1000	8000
**Gaussian mutation rate**	0.2	0.2
**Mutation usage rate**	1.0	1.0
**Number of runs**	30	30
**Crossover type**	Uniform	Uniform
**Crossover usage rate**	1.0	1.0

**Table 4:** Coded keys for AM technique and part feature attributes.

Coded Key	AM Technique	Material Type	Surface Finish	Build Speed	Feature Resolution
1	LOM 1	Polymer	Smooth	Slow	Fine Detail
2	LOM 2	Metal	Medium	Medium	Medium
3	FDM 1	Ceramic	Coarse	Fast	Coarse
4	FDM 2	Paper			
5	SLS 1	Composite			
6	SLS 2				
7	SLA 1				
8	SLA 2				
9	3DP 1				
10	3DP 2				

**Table 5:** Average identification accuracy for 100 and 300 datasets.

Method	Average Accuracy	Best Accuracy
Identification accuracy for 100 datasets		
Baseline	N/A	10.0%
GEFeW	70.0%	80.0%
Identification accuracy for 300 datasets		
Baseline	N/A	40.0%
GEFeW (SSGA)	97.33%	100.0%

**Table 6:** Average weights of GEFeW for AM classification for 100 datasets.

Feature	Material	Length (mm)	Width (mm)	Height (mm)	Build Volume (mm^3^)	Min. Thickness (mm)	Surface Finish (µm)	Build Speed (mm/h)	Feature Resolution (µm)
Weight	0.8282	0.0078	0.0004	0.0031	0.0	0.0645	0.63432	0.8276	0.76555

**Table 7:** The frequency of correct classifications for each AM classification for 100 datasets.

LOM2	LOM1	FDM2	FDM1	SLS2	SLS1	SLA2	SLA1	3DP2	3DP1
30	30	1	29	1	30	29	30	0	30

**Table 8:** Design rule ranges for different AM processes.

	SLA	SLS	FDM	3DP	LOM
Build speed (mm/h)	7 to 30	25 to 60	7 to 43	20 to 50	40 to 150
Surface finish (µm)	80 to 130	125 to 250	150 to 300	180 to 300	200 to 500
Resolution (µm)	35 to 100	60 to 150	200 to 400	50 to 180	100–300 (paper) and 150–300 (plastic)

**Table 9:** ANOVA for evaluating algorithms.

SUMMARY						
Groups	Count	Sum	Average	Variance		
SSGA	30	292	97.33	0.34		
EDA	30	280	93.33	0.44		
PSO	30	274	91.33	0.60		
						
ANOVA						
Source of Variation	SS	df	MS	F	*p*-value	F crit
Between Groups	5.6	2	2.8	6.09	0.0033	3.10
Within Groups	40	87	0.46			
Total	45.6	89				

**Table 10:** Average weights of GEFeW for AM classification for 300 datasets.

Feature	Material	Length (mm)	Width (mm)	Height (mm)	Build Volume (mm^3^)	Min. Thickness (mm)	Surface Finish (µm)	Build Speed (mm/h)	Feature Resolution (µm)
Weight	0.684	0.003435	0.00223	0.08627	0.040351	0.23344	0.388319	0.78108	0.2250195

**Table 11:** Frequency of correct classifications for each AM classification for 300 datasets.

LOM1	LOM2	FDM1	FDM2	SLS1	SLS2	SLA1	SLA2	3DP1	3DP2
30	30	30	30	30	28	30	30	30	24

## Data Availability

The data presented in this study are available upon request from the corresponding author. The data are not publicly available due to confidential part designs used in this research.
